# Annual program review process: an enhanced process with outcomes

**DOI:** 10.1080/10872981.2018.1527626

**Published:** 2018-10-11

**Authors:** Marcy Wiemers, Mark Nadeau, James Tysinger, Cristian Fernandez Falcon

**Affiliations:** Department of Family and Community Medicine, UT Health San Antonio, San Antonio, TX, USA

**Keywords:** Annual program review of educational effectiveness, annual program review, family medicine residency, accreditation council for graduate medical education, ACGME, graduate medical education(GME)

## Abstract

The Accreditation Council for Graduate Medical Education’s required Annual Program Review of Educational Effectiveness (APREE) has helped us improve our program and change its culture to one of continuous quality improvement. This report outlines our systematic process and describes specific outcomes it has produced over a 10-year period. We identified ways to enhance our APREE after reading articles that described various ways to conduct the process found in a PubMed and OvidSP search and relevant policies from our local Graduate Medical Education Office. After discussing options, we incorporated new ideas into our APREE and tasked our Program Evaluation Committee to track outcomes from objectives developed by faculty and residents during each APREE. Objectives from faculty and residents in 10 years of our APREE led to major improvements (e.g., increased board pass rate) in our program. In addition, the enhanced APREE process gradually changed our residency’s culture to one that embraces continuous quality improvement. A systematic APREE process can engage residents and faculty in improving specific components of a residency. Besides providing outcomes for Web Ads and Self-Study items, the APREE models quality improvement techniques to residents, involves a wide array of stakeholders, and helps program stakeholders embrace continuous quality improvement.

## Introduction

For over a decade, Accreditation Council for Graduate Medical Education (ACGME) and institutional policies required residencies to conduct an Annual Program Review of Educational Effectiveness (APREE) [1–3]. Conducted and monitored by the Program Evaluation Committee (PEC), the APREE is intended to be a process improvement activity that helps programs evolve and improve[4]. Process improvement and leadership are essential in managing graduate medical education curricula, demonstrated by new requirements, the New Accreditation System including the 10-year Self-study and the introduction of the Milestones [5–7]. Detailed guidance on conducting APREEs from the ACGME and institutional Graduate Medical Education Offices is helpful[2], and authors have described how they use it to conduct their reviews [8–14]. While our APREE met the requirement, the faculty and residents failed to fully embrace the process because they did not recognize their suggestions produced meaningful change. This paper describes the APREE process our program implemented to engage stakeholders in program ownership and implement program improvements that fit with the ACGME’s pursuit of excellence [15,16].

## Methods

The UT Health San Antonio Family Medicine Residency is an academic-based program with clinical sites supported by University Health System, which manages CareLink (a financial assistance program for patients who lack health insurance), and the South Texas Veterans Health Care System (STVHCS). We have 15 residents per class, 31% of who are US graduates, and a core faculty of 20 physicians who also teach medical students.

We used two strategies to improve our APREE process. One, we searched PubMed and OvidSP for articles using these terms: Annual Program Review, Program Review Residency Process Improvement, Program Review Medical Process Improvement, and other combinations of these words. The initial literature search, conducted for the 2007 article (Nadeau, 2007), made us realize that describing our APREE process with outcomes would be useful to others. For this article, we expanded our review to include articles published after 2007. These recently published articles described how other programs conducted their APREE and the outcomes their process produced, but they gave limited information about best practices. Reviewing the program improvements associated with our process made us aware that our process had elements of strategic planning coupled with continuous quality improvement. We communicated this perspective to the residents and faculty in our next APREE. Second, we followed our Designated Institutional Official’s (DIO) policies for conducting the APREE [2,3]. We incorporated these two activities into our enhanced process[1].

Our APREE involves these steps:

1. Staff blocks the half-day APREE meeting on resident and faculty calendars.
2. Chiefs and class representatives summarize anonymous resident evaluations and a curricular review during a PEC meeting.
3. PEC members specify residency components to address in online surveys that faculty and residents complete.
4. Faculty and residents complete the surveys by rating residency components, explaining ratings, and commenting on topics not included. (Note: Shortening surveys’ length increased completion rates and focused on issues that needed improvement. All responses are anonymous and confidential.)
5. Program leaders analyze faculty and resident ratings and narratives to identify program components to present in the APREE.
6. A faculty member starts the APREE meeting by presenting its agenda, stating last year’s objectives and outcomes to reaffirm the meeting’s value, highlighting major accomplishments, describing findings from faculty and resident surveys, and leading the group to complete a strengths, weaknesses, opportunities, and threats (SWOT) analysis.
7. Faculty and residents select five to six most pertinent issues from surveys to discuss in small groups.
8. Participants in the small groups draft action items after discussion of the identified issue.
9. A spokesperson from each small group presents the group’s findings and action items.
10. The large group discusses and votes on four to six action items for the coming year. (Staff records the small and large group discussions for the PEC.)
11. The PEC finalizes the APREE by:

- Reviewing the small and large group discussions, the SWOT analysis, and items on the action plan.
- Developing a durable SWOT document to ensure the program complies with and documents all ACGME requirements for improvement throughout the year.
- Stating SMART goals for the coming year using this SWOT analysis, developing an action plan for implementing the goals, discussing how the action plan aligns with the Self Study, and evaluating the usefulness of this document to align with future requirements to include the program description and AIMS.

## Results

Each year, our APREE process produces three products. First, a SWOT analysis from the group and PEC (see Table 1) provides an ongoing assessment of our strengths, weaknesses, opportunities, and threats. Engaging the residents and faculty in a SWOT analysis during each APREE helped them see the APREE as part of a strategic process of systematic improvement. Discussing our program’s strengths, weaknesses, opportunities, and threats made them reflect on how previous APREEs produced goals and activities that improved specific educational, administrative, and clinical aspects of our program. Thus, stakeholders saw the value in actively engaging in each APREE since their contributions helped advance our residency. Second, SMART goals for the year help focus the residency on specific improvements. Third, an action plan with outcomes in three categories (e.g., educational, clinical, and administrative) (see Table 2) from the SMART goals improves the program and reinforces the process’ value to stakeholders. The products are created from and used by the events in the timeline in Table 3 surrounding the APREE.

**Table 1.** SWOT analysis with action plan example for the 2017–2018 academic year.

Item	Strategy	Resources	Timeline	Action/Metric
**Strengths**				
Community involvement in the management of chronic pain	Offer chronic pain support groups in collaboration with community partners [e.g., the Area Health Education Center (AHEC)]	Existing residency team members (e.g., community health workers, social workers, behavior health therapist)	April 2017–January 2018	Number of patients participating in chronic pain support groups
**Weaknesses**				
No point of care testing in the Family Health Center (FHC)	Increase opportunities for learning point of care testing Acquire microscopes	Women’s Clinic Skin Clinic Excision Clinic	April 2017–March 2018	Document competency in point of care testing in all residents before graduation
**Opportunities**				
Practice management experience in coding	One-on-one coaching in coding by faculty	Residency faculty	April 2017–January 2018	Residents must code 4 of 5 patient notes according to CMS standard
**Threats**				
Decreased number of women’s health procedures in long acting reversible contraception (LARC) in the FHC	Attract more female patients with insurance	FHC is the largest clinic in the system Faculty are trained in LARC procedures	April 2017–April 2018	Document number of LARC procedures performed by graduating residents

**Table 2.** Examples of clinical, educational, and administrative outcomes over 10 years of annual program reviews.

Academic Year	Examples
2007–2008	Clinical: Increased pediatric patient volume and exposure
2008–2009	Educational: Improved EKG training
2009–2010	Educational: Instituted resident support groups
2010–2011	Educational: Increased walking rounds on Inpatient Service
2011–2012	Administrative: Improved resident parking at University Hospital
2012–2013	Clinical: Increased patient volume on services (e.g., pediatric, obstetrical) by integrating rotations
2013–2014	Administrative: Increased faculty development in patient safety
2014–2015	Educational: Provided residents with travel funds when presenting at regional and national conferences
2015–2016	Clinical: Increased experiences in pediatric urgent/emergency care
2016–2017	Educational: Increased number of long-acting reversible contraception (LARC) and gynecological procedures
2017–2018	Administrative: Improved documentation of faculty development

**Table 3.** Timeline and milestones for the program’s annual program review of educational effectiveness.

Month	Supporting activity
January–February	Gather all program evaluation data (e.g., ABFM pass rate, compile resident evaluations on rotations from New Innovations)
March	Chief Residents and Class Representatives summarize rotation evaluations to identify potential items for resident and faculty surveys Program Evaluation Committee (PEC) develops residency and faculty surveys
April	Resident and faculty surveys are entered into SurveyMonkey and sent to individual residents and faculty Resident and faculty survey responses are compiled by a faculty member outside the residency Program leaders analyze data to present in the APREE
May	The APREE occurs during the middle of this month Data (i.e., SWOT analysis, goals and action plan for the next year) from the APREE are compiled by the faculty member who facilitates the APREE and forwarded to the PEC chair The PEC reviews data from the APREE and confirms program agreed upon SWOT and measurable goals identified for our action plan
June	The PEC chair confirms all documents including SWOT and action plan are updated before the new academic year
July	The program director educates our incoming residents about our APREE and that it relies heavily on the residents’ completing rotation evaluations
August	The PEC reviews progress on measurable goals and reevaluates how they fit into our AIMS and self-study
November	The PEC confirms progress on measurable goals
December	Residents are reminded via email to complete rotation evaluations and inform the chief residents and/or residency leaders about any praises or concerns about educational venues, quality, or needs

**Figure 1.**
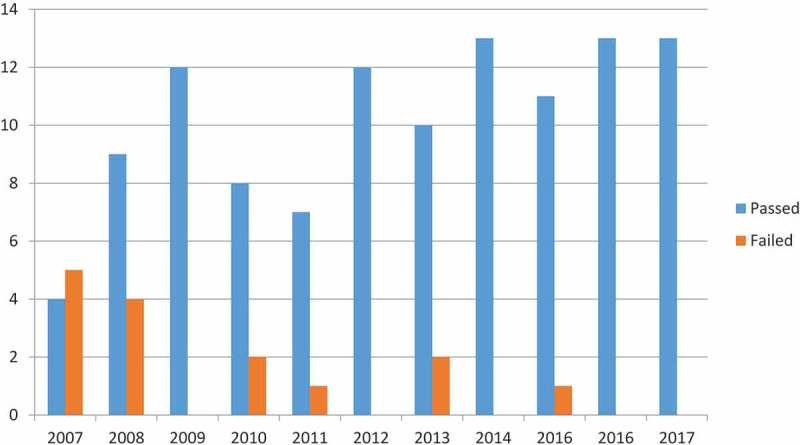
UTHSCSA family medicine residency graduates’ ABFM certification examination pass and failure rates.

Two major program outcomes illustrate the process’ value: Scholarly activity and ABFM Certification Examination pass rates. In the 5 years before our first APREE, our residents presented rarely at regional and national conferences. Since faculty and residents identified scholarly activity as a concern in 2010, there was purposeful quality improvement surrounding this issue focused on mentorship, venues, and financial support. Following this process, residents, currently on average, present five posters at the national NAPCRG conference. This has also produced, on average, 12 resident contributed 5-min Clinical Consult book chapters. We have residents presenting at over five different venues each year. Focusing on the ABFM Certification Examination pass rates from several APREEs produced significant improvements (see Figure 1). Multiple activities were implemented before new ACGME requirements were met over the studied period [1,17,18]. This included interactive didactics focused on common board issues, incorporating board review questions in multiple arenas, aggressive monitoring and learning plans based on in training exam scores, and third-year attendance of a board review course. Since many variables impact resident performance on the ABFM Certification Examination, we believe, but cannot prove, that the activities instituted from our APREE positively influenced our presently favorable pass rate. The increase pass rate is statistically significant with a *p* value of 0.00007;however, more data in the future will help support causality.

Finally, the APREE process has changed our program’s culture to one of continuous improvement. ‘Begin with the end in mind’, the recommended maxim from the 7 Habits of Highly Effective People, is especially apt here[19]. Initially, faculty and residents participated because they saw the APREE as a requirement. Faculty and residents fully engage and take the process seriously after seeing their efforts improve the program.

## Discussion

Our APREE now allows us to openly discuss our strengths, weaknesses, opportunities, and threats and plan changes that will improve our program. Engaging stakeholders in the process and discussing the impact of changes models quality improvement and makes faculty and residents value participating. Many programs can benefit from an expanded view of the APREE process. Established programs with few identified issues can increase dialogue with all stakeholders and enhance established processes to produce continued improvements even in strong areas. For example, scholarly activity among our residents met the ACGME standard, but is now an area of excellence after we instituted faculty development, mentorship, and support following one APREE. New programs or those with multiple resident concerns can achieve many improvements by giving faculty and residents ownership in this APREE process. Modeling the desire for continuous improvement and showing stakeholders their suggestions are embraced can quickly change a program’s culture and climate.

Improvements in a residency vary in complexity and the extent to which they resolve an issue. Some issues can be addressed quickly when programs control the issue at hand. For example, two of our faculty began consistently teaching an electrocardiogram workshop after residents expressed concerns about reading EKGs. However, a program’s lack of complete control may impair its ability to quickly resolve an issue. For example, our health system obtained a grant for long-acting reversible contraception (LARC) for indigent patients, but did not include family physicians among those authorized to use the grant money for LARCs. We are presently collaborating with the health system to address this educational and clinical issue. Faculty engagement develops bonds with faculty in other departments, resident engagement provides insight into opportunities and resources in other departments, and administrative engagement presents clinical and educational needs in the family health center within the context of the system.

A program may never completely resolve an issue (e.g., improved parking) due to system limitations, but residents can recognize efforts and improvements. After residents expressed safety (i.e., lots were dark and far from the hospital) and wellness (i.e., searching for a parking slot was stressful in the mornings) concerns about the lack of parking at our hospital in an APREE, program leaders tried to improve the situation. Since resident safety and wellness are top priorities in our program, leaders agreed that solving the parking concerns would enhance resident safety and wellness. Program leaders described residents’ concerns with our department chair, medical school officials, and health system personnel (e.g., police) to provide more parking slots closer to the hospital. Constraints (e.g., number of parking slots) limited completely resolving the residents’ concerns, but measures like police escorts and parking slots open at night, addressed some concerns. Leaders’ routinely reporting progress on parking satisfied most residents and showed them we were committed to resolving the parking issue long term. Since needs can change, other issues may resolve with reassessment (e.g., residents reported less interest in support groups when wellness initiatives increased). With this transparency, we do not use energy on issues that stakeholders no longer value. When department resources alone fail, system resources (e.g., training faculty in patient safety initiatives) can remedy some needs.

Our APREE process tracks and continuously improves areas that reaffirm our AIMS, creating safe, reliable, patient-centered, and data-driven family medicine physicians. Each year, we use 4 hours during our Wednesday afternoon didactic period to conduct the APREE. Residents are required to attend didactics, so the APREE does not decrease their clinical activity. Faculty usually attend unless they are on vacation or ill, so faculty participation in the APREE accounts for a loss of about 40 clinical hours. We think engaging residents and faculty at the same time makes the process more transparent and allows the residents to hear what faculty think about an issue without having to discuss concerns with individual faculty. Residency leaders prefer the present process because it facilitates documentation and helps meet an important ACGME and local institutional requirement.

Planning for the APREE consumes some time. A meeting to agree on items for the resident and faculty surveys takes about 6 hours. Revising and distributing the surveys on Survey Monkey and compiling the resident and faculty responses take 4 hours of a faculty member’s time. Placing these responses on Canvas, our institution’s educational management system, takes less than 10 minutes of staff time. Stakeholders can access, download, and bring faculty and resident responses to the meeting, so there is no expense for photocopying or distributing responses.

## Presentations

Nadeau MT, Tysinger J. The Required Annual Program Review: A Strategic Process with Outcomes. A paper presented at the 42nd Annual Workshop for Directors of Family Medicine Residencies, Association of Family Medicine Residency Directors, Kansas City, MO, June 2010.

Nadeau MT, Tysinger J. The Required Annual Program Review: A Strategic Process with Outcomes. A paper presented at the STFM 43rd Annual Spring Conference, Vancouver, British Columbia, April 2010.

## Article

We also published an early version of our process with outcomes in Family Medicine (see citation below). The article we are now submitting details updates in the process, specifies expanded outcomes in categories, and describes how the process changed our program’s culture.

Nadeau MT, Tysinger JW. The annual program review of effectiveness: A process improvement approach. Fam Med, 2102;44(1):32–38.
